# Introduction to the human gut microbiota

**DOI:** 10.1042/BCJ20160510

**Published:** 2017-05-16

**Authors:** Elizabeth Thursby, Nathalie Juge

**Affiliations:** The Gut Health and Food Safety Programme, Institute of Food Research, Norwich Research Park, Norwich NR4 7UA, U.K.

**Keywords:** gastrointestinal tract, gut microbiota, symbiosis

## Abstract

The human gastrointestinal (GI) tract harbours a complex and dynamic population of microorganisms, the gut microbiota, which exert a marked influence on the host during homeostasis and disease. Multiple factors contribute to the establishment of the human gut microbiota during infancy. Diet is considered as one of the main drivers in shaping the gut microbiota across the life time. Intestinal bacteria play a crucial role in maintaining immune and metabolic homeostasis and protecting against pathogens. Altered gut bacterial composition (dysbiosis) has been associated with the pathogenesis of many inflammatory diseases and infections. The interpretation of these studies relies on a better understanding of inter-individual variations, heterogeneity of bacterial communities along and across the GI tract, functional redundancy and the need to distinguish cause from effect in states of dysbiosis. This review summarises our current understanding of the development and composition of the human GI microbiota, and its impact on gut integrity and host health, underlying the need for mechanistic studies focusing on host–microbe interactions.

## Introduction

The human gastrointestinal (GI) tract represents one of the largest interfaces (250–400 m^2^) between the host, environmental factors and antigens in the human body. In an average life time, around 60 tonnes of food pass through the human GI tract, along with an abundance of microorganisms from the environment which impose a huge threat on gut integrity [1]. The collection of bacteria, archaea and eukarya colonising the GI tract is termed the ‘gut microbiota’ and has co-evolved with the host over thousands of years to form an intricate and mutually beneficial relationship [2,3]. The number of microorganisms inhabiting the GI tract has been estimated to exceed 10^14^, which encompasses ∼10 times more bacterial cells than the number of human cells and over 100 times the amount of genomic content (microbiome) as the human genome [2,4]. However, a recently revised estimate has suggested that the ratio of human:bacterial cells is actually closer to 1:1 [5]. As a result of the vast number of bacterial cells in the body, the host and the microorganisms inhabiting it are often referred to as a ‘superorganism’ [4,6].

The microbiota offers many benefits to the host, through a range of physiological functions such as strengthening gut integrity or shaping the intestinal epithelium [7], harvesting energy [8], protecting against pathogens [9] and regulating host immunity [10]. However, there is potential for these mechanisms to be disrupted as a result of an altered microbial composition, known as dysbiosis. With increasingly sophisticated methods to profile and characterise complex ecosystems being developed, a role for the microbiota in a large number of intestinal and extra-intestinal diseases has become steadily apparent [11,12]. This review summarises our current understanding of the development and composition of the human GI microbiota, and its impact on gut integrity and host health.

## Composition and structure of the human GI microbiota

Around a decade ago, most knowledge about the adult human gut microbiota stemmed from labour-intensive culture-based methods [13]. Recently, our ability to survey the breadth of the gut microbiota has greatly improved due to the advent of culture-independent approaches such as high-throughput and low-cost sequencing methods. Targeting of the bacterial 16S ribosomal RNA (rRNA) gene is a popular approach [14,15], since this gene is present in all bacteria and archaea and contains nine highly variable regions (V1–V9), which allows species to be easily distinguished. Former techniques concentrated on sequencing the entire 16S rRNA gene. In an early study using this method, the extreme insensitivity and bias of culturing methods were highlighted, since 76% of the rRNA sequences obtained from an adult male faecal sample belonged to novel and uncharacterised species [16]. Recently, the focus of 16S rRNA sequencing has shifted to analysing shorter subregions of the gene in greater depth [15]; however, the utilisation of shorter read lengths can introduce errors [14]. More reliable estimates of microbiota composition and diversity may be provided by whole-genome shotgun metagenomics due to the higher resolution and sensitivity of these techniques [14]. Combined data from the MetaHit and the Human Microbiome Project have provided the most comprehensive view of the human-associated microbial repertoire to date [17,18]. Compiled data from these studies identified 2172 species isolated from human beings, classified into 12 different phyla, of which 93.5% belonged to Proteobacteria, Firmicutes, Actinobacteria and Bacteroidetes. Three of the 12 identified phyla contained only one species isolated from humans, including an intestinal species, *Akkermansia muciniphila*, the only known representative of the Verrucomicrobia phyla. In humans, 386 of the identified species are strictly anaerobic and hence will generally be found in mucosal regions such as the oral cavity and the GI tract [17].

The gut microbiota is not as diverse as the microbial communities from some other bodily sites and reveals a high degree of functional redundancy [19–21]. An extensive catalogue of the functional capacity of the human gut microbiome was recently obtained, where 9 879 896 genes were identified through a combination of 249 newly sequenced and 1018 published samples [18]. The study identified the presence of country-specific microbial signatures, suggesting that gut microbiota composition is shaped by environmental factors, such as diet, and possibly also by host genetics [18]. However, it should also be noted that microbiotas that differ in terms of composition may share some degree of functional redundancy, yielding similar protein or metabolite profiles [22]. This information is crucial for developing therapeutic strategies to modify and shape the microbial community in disease.

## Development of the human GI microbiota

The development of the microbiota is generally believed to begin from birth, although this dogma is challenged by a limited number of studies in which microbes were detected in womb tissues, such as the placenta [23,24]. After birth, the GI tract is rapidly colonised, with life events such as illness, antibiotic treatment and changes in diet causing chaotic shifts in the microbiota [24,25]. The mode of delivery also appears to affect the microbiota composition, with vaginally delivered infants' microbiota containing a high abundance of lactobacilli during the first few days, a reflection of the high load of lactobacilli in the vaginal flora [26,27]. In contrast, the microbiota of infants delivered by C-section is depleted and delayed in the colonisation of the *Bacteroides* genus, but colonised by facultative anaerobes such as *Clostridium* species [28,29]. Whilst the faecal microbiota of 72% of vaginally delivered infants resembles that of their mothers' faecal microbiota, in babies delivered by C-section, this percentage is reduced to only 41% [30]. In early stages of development, the microbiota is generally low in diversity and is dominated by two main phyla, Actinobacteria and Proteobacteria [24,31]. During the first year of life, the microbial diversity increases and the microbiota composition converges towards a distinct adult-like microbial profile with temporal patterns that are unique to each infant [32]. By around 2.5 years of age, the composition, diversity and functional capabilities of the infant microbiota resemble those of adult microbiota [24,25]. Although, in adulthood, the composition of the gut microbiota is relatively stable, it is still subject to perturbation by life events [33]. In individuals over the age of 65, the microbial community shifts, with an increased abundance of Bacteroidetes phyla and *Clostridium* cluster IV, in contrast with younger subjects where cluster XIVa is more prevalent [34]. In contrast, a separate study observed that the microbiota of a young cohort and an elderly population (70 years) were relatively comparable, whilst the diversity of the microbiota from a cohort of centenarians was significantly reduced [35]. The centenarian microbiota also exhibited group-specific differences such as an increase in the abundance of facultative anaerobes (e.g. *Escherichia coli*) and rearrangement of the profile of butyrate producers (e.g. decrease in *Faecalibacterium prausnitzii*) [35]. In the elderly population, a significant relationship has been identified between diversity and living arrangements, such as community dwelling or long-term residential care [36]. Overall, the capacity of the microbiota to carry out metabolic processes such as short-chain fatty acid (SCFA) production and amylolysis is reduced in the elderly, whilst proteolytic activity is increased [37]. Given the increasing evidence for the role of SCFAs as key metabolic and immune mediators (as reviewed below), it was postulated that the decrease in SCFAs may nurture the inflamm-ageing process in the intestine of aged people [38].

## Biogeography of the human microbiota in the GI tract

Microbiota composition in the GI tract is reflective of the physiological properties in a given region and is stratified on both a transverse and longitudinal axis [39]. The density and composition of the microbiota are affected by chemical, nutritional and immunological gradients along the gut. In the small intestine, there are typically high levels of acids, oxygen and antimicrobials, and a short transit time [40]. These properties limit bacterial growth, such that only rapidly growing, facultative anaerobes with the ability to adhere to epithelia/mucus are thought to survive [40]. In mice, the small-intestine microbial community is largely dominated by Lactobacillaceae [41]. In contrast, colonic conditions support a dense and diverse community of bacteria, mainly anaerobes with the ability to utilise complex carbohydrates which are undigested in the small intestine. In the colon Prevotellaceae, Lachnospiraceae and Rikenellaceae have been shown to dominate [40,41].

In contrast with the differing microbiota composition between varying GI organs, the microbiota of different colorectal mucosal regions within the same individual is spatially conserved in terms of both composition and diversity [42,43]. This feature is apparent even during periods of localised inflammation [43]. On the other hand, the faecal/luminal and mucosal compositions are significantly different [42,43]. For example, the abundance of Bacteroidetes appears to be higher in faecal/luminal samples than in the mucosa [42,44]. In contrast, Firmicutes, specifically *Clostridium* cluster XIVa, are enriched in the mucus layer compared with the lumen [44]. Interestingly, recent experiments in mice colonised with a diverse specific pathogen-free microbiota showed that the outer mucus of the large intestine forms a unique microbial niche and that bacterial species present in the mucus show differential proliferation and resource utilisation compared with the same species in the intestinal lumen [45]. These observations highlight the need for careful consideration in choosing a sampling method when analysing the microbiota composition.

Inter-individual differences in the species and subspecies arrangement are proposed to outweigh differences in the community arrangement within an individual [42,46,47]. Suggestions have been made of the presence of a ‘core microbiota’, proposed to be a set of the same abundant organisms present in all individuals. However, more similarity can be observed in the repertoire of microbial genes present between individuals than the taxonomic profile, suggesting that the core microbiota may be better defined at a functional rather than organism level [46]. Recently, it has been possible to classify individual microbiota arrangements into ‘community types’ that can be predictive of each other and are associated with background [48]. Multi-dimensional analysis of 33 samples from different nationalities revealed the presence of three enterotypes identifiable by variations in the level of one of three genera: *Bacteroides* (enterotype 1), *Prevotella* (enterotype 2) and *Ruminococcus* (enterotype 3) [49]. However, evidence surrounding the existence and formation of these enterotypes is controversial, as thoroughly reviewed in [50].

## Factors shaping the GI microbiota

The microbiota composition is subject to shaping by host and environmental selective pressures. To protect from injury and maintain homeostasis, the GI tract limits exposure of the host immune system to the microbiota by recruitment of a multifactorial and dynamic intestinal barrier. The barrier comprises several integrated components including physical (the epithelial and mucus layers), biochemical (enzymes and antimicrobial proteins) and immunological (IgA and epithelia-associated immune cells) factors [51]. An individual microbe's longevity is determined by whether it is contributing to the range of essential functions on which host fitness relies. It is proposed that organisms who do not contribute beneficial functions are controlled by, and may occasionally be purged during, for example, transferral of the microbiota to a new host [52,53].

Gut microbes must be adapted to a certain type of lifestyle due to the relatively fewer number of biochemical niches available in the gut, compared with other microbial-rich environments. In the gut, energy can generally be derived through processes such as fermentation and sulphate reduction of dietary and host carbohydrates. The organisms that can survive in the gut are therefore limited by their phenotypic traits [52].

Current research suggests that diet exerts a large effect on the gut microbiota [40]. Meta-transcriptomic studies revealed that the ileal microbiota is driven by the capacity of the microbial members to metabolise simple sugars, reflecting adaptation of the microbiota to the nutrient availability in the small intestine [54]. Shaping of the colonic microbiota is subject to the availability of microbiota-accessible carbohydrates (MACs) that are found in dietary fibre. Extreme ‘animal-based’ or ‘plant-based’ diets result in wide-ranging alterations of the gut microbiota in humans [55]. The influence of fibre was demonstrated in a crossover study showing that otherwise matched diets high in resistant starch or in non-starch polysaccharide fibre (wheat bran) resulted in the strong and reproducible enrichment of different bacterial species in the human gut [56]. Feeding methods can also affect the abundance of some bacterial groups in the gut microbiota of infants. For example, fucosylated oligosaccharides present in human milk can be utilised by *Bifidobacterium longum* and several species of *Bacteroides* allowing them to outcompete other bacteria such as *E. coli* and *Clostridium perfringens* [57,58]. Whilst the abundance of *Bifidobacterium* spp. in breast-fed infant microbiota is typically high [57], this is reduced in formula-fed infants [59]. Furthermore, formula-fed infant microbiota has an increased diversity and altered levels of other groups such as *E. coli*, *Clostridium difficile*, *Bacteroides fragilis* and lactobacilli [59–61]. The microbiota of undernourished infants is immature, dysbiotic and contains greater numbers of enteropathogens, such as Enterobacteriaceae [62]. Infants from rural Africa, with a diet dominated by starch, fibre and plant polysaccharides, harbour a microbiota that is abundant in the Actinobacteria (10.1%) and Bacteroidetes (57.7%) phyla [63]. In contrast, in European children, whose diet is rich in sugar, starch and animal protein, the abundance of these groups is reduced to just 6.7 and 22.4% [63]. Some SCFA producers, such as *Prevotella*, were exclusive to the microbiota of African children [63]. This trend was also apparent in healthy individuals consuming high amounts of carbohydrates and simple sugars [64]. A decreased SCFA output is also evident in individuals consuming a low MAC diet, a notable effect since SCFAs play an important role in host health via, for example, anti-inflammatory mechanisms [65]. The abundance of MACs is substantially reduced in the Western diet. Administration of a low MAC diet to mice results in a reduction in microbial diversity [66]. Restoration of diversity requires administration of MACs in combination with the bacterial taxa that were missing [66]. Recent studies using gnotobiotic mice showed that certain microbial species can be used to restore growth impairments transmitted by microbiota from malnourished children, raising the possibility of using these species as a therapeutic intervention to counteract the negative effects of undernutrition [67–69].

Intestinal mucus also provides a source of carbohydrates to the gut microbiota [70,71]. The intestinal mucus layers are built around the large highly glycosylated gel-forming mucin MUC2 (Muc2 in mouse) secreted by goblet cells [72]. The glycan structures present in mucins are diverse and complex and based on four core mucin-type *O*-glycans containing *N*-acetylgalactosamine, galactose and *N*-acetylglucosamine. These core structures are further elongated and frequently terminated by fucose and sialic acid sugar residues. Collectively, *O*-glycans account for up to 80% of the total molecular mass of Muc2/MUC2 [73]. Mucus is present throughout the GI tract and is thickest in the colon where it is crucial in mediating the host–microbiota relationship [74]. Normalisation of host intestinal mucus layers requires long-term microbial colonisation [75]. Colonic mucus is divided into two layers consisting of a dense and impermeable inner layer and a loose outer coating which is penetrable by bacteria [74]. Whilst the inner layer is virtually sterile, in the outer layer, the mucin proteins, which are decorated by a rich and diverse repertoire of *O*-glycans, provide an energy source and preferential binding sites for commensal bacteria [73,76,77]. The arrangement of the outer mucus layer provides a unique niche in which bacterial species display different patterns of proliferation and utilisation of resources compared with their counterparts in the lumen [45]. The type of mucin O-glycosylation is dependent on the glycosyltransferases expressed and where in the Golgi apparatus they are located [71], alterations of which affect the microbiota composition. For example, the presence (secretor) or absence (non-secretor) of H and ABO antigens in GI mucosa, determined by the FUT2 genotype (a gene expressing an α1,2-fucosyltransferase), affects the abundance of many bacterial groups [78]. Mucus and mucin glycosylation are therefore key in shaping the microbiota and allow for the selection of the most optimal microbial species to mediate host health [79–81]. A depletion of MACs from the diet of mice can result in thinner mucus in the distal colon, increased proximity of microbes to the epithelium and heightened expression of the inflammatory marker REGIIIβ [82]. The erosion of the colonic mucus barrier under dietary fibre deficiency is associated with a switch of the gut microbiota towards the utilisation of secreted mucins as a nutrient source [83]. On the other hand, administration of a mucin degrader, *A. muciniphila*, to mice prevents the development of high-fat diet-induced obesity and ameliorates metabolic endotoxemia-induced inflammation through restoration of the gut barrier [84,85]. Some of these effects are the result of increased mucin secretion and intestinal tight-junction proteins, which highlight the dynamic role played by mucin degraders in their interaction with the host. The protective role of *A. muciniphila* could be recapitulated using *A. muciniphila* purified membrane protein or the pasteurised bacterium [86]. Recently, *A. muciniphila* supplementation was shown to significantly alleviate body weight gain and reduce fat mass in chow diet-fed mice by relieving metabolic inflammation [87]. These studies suggest the potential of *A. muciniphila* as a therapeutic option to target human obesity and associated disorders.

The capacity of gut bacteria to utilise dietary or mucin glycans is dictated by the repertoire of glycoside hydrolases (GHs) and polysaccharide lyases (PLs) encoded by their genomes [70]. Some species act as generalists able to degrade a wide range of polysaccharides, whilst others are specialists targeting specific glycans [88]. Bacteroidetes encode many more glycan-cleaving enzymes (137.1 GH and PL genes per genome) than Firmicutes members (39.6 GH and PL genes per genome) [89]. The *Bacteroides thetaiotaomicron* genome contains 260 GHs, in comparison with the 97 hydrolases encoded by humans [90]. The GH13 family, which contains enzymes involved in the breakdown of starch, is the most represented family in the gut microbiota [89]. Recently, the detailed biochemical and structural characterisation of the extensive degrading apparatus of prominent gut species such as *B. thetaiotaomicron* or *Bacteroides ovatus* revealed that the recognition and breakdown of complex carbohydrates, such as xylan, mannan, xyloglucan or starch, by the human gut microbiota is significantly more complex than previously suggested [91–96]. Although less studied, members of Firmicutes also show some unique and complex features such as the recent discovery of amylosomes in the resistant starch-utilising bacterium *Ruminococcus bromii* [97].

Diversification of the microbial population can occur through, for example, mutation or lateral gene transfer [98,99]. The introduction of new bacterial functions promotes niche variation, creating a positive feedback loop in which more diversification can occur [100,101]. Co-operation between gut microbes also allows colonisation by a more diverse set of organisms, shaping the gut microbiota community. Microbial cross-feeding is one mechanism proposed to mediate this effect. Some carbohydrate fermentation products, including lactate, succinate and 1,2-propanediol, do not usually accumulate to high levels in the human colon of healthy adults, as they can serve as substrates for other bacteria, including propionate and butyrate producers [102]. For example, acetate produced by fermentation of resistant starch by *R. bromii* [103] or lactate produced by lactic acid bacteria such as lactobacilli and bifidobacteria provides substrate for other microbiota members such as *Eubacterium hallii* and *Anaerostipes caccae* which convert it into butyrate [104,105]. Recently, *B. ovatus* has been demonstrated to perform extracellular digestion of inulin at its own cost, but at an advantage to other species which provide reciprocal benefits [106]. Such co-operation is particularly apparent in the outer mucus layer where mucin-degrading bacteria provide mono- or oligosaccharides to bacteria without specialised mucolytic capability [45]. For example, the capacity of cleaving sialic acid off mucins is restricted to bacterial species encoding GH33 sialidases. Many bacteria, including pathogens such as *Salmonella* Typhimurium or *C. difficile*, lack a sialidase but harbour a ‘nan cluster’ dedicated to sialic acid metabolism, and thus rely on other members of the gut microbiota to provide them with this source of carbon [107]. Intramolecular *trans*-sialidase is a new class of sialidases recently identified in *Ruminococcus gnavus* strains that may play a role in the adaptation of gut commensal bacteria to the mucosal niche [70,108,109]. This activity may provide such bacteria with a competitive nutritional advantage over other species within the gut mucosal environment, specifically in inflammatory bowel diseases, which is rich in short, sialylated mucin glycans [70,110].

The availability of sulphated compounds in the colon, either of inorganic (e.g. sulphates and sulphites) or organic (e.g. dietary amino acids and host mucins) origin, can influence specific groups of bacteria such as sulphate-reducing bacteria, which are residents of the gut microbiota that have been implicated in the aetiology of intestinal disorders such as IBD, IBS or colorectal cancer [111].

The distribution of bile acids in the small and large intestine can also affect the bacterial community dynamics in the gut as thoroughly reviewed [112,113]. Primary bile acids, such as taurocholate, can provide homing signals to gut bacteria and promote germination of spores, and may also facilitate recovery of microbiota after dysbiosis induced by antibiotics or toxins [114]. Furthermore, reduced bile acid concentration in the gut may play an important role in allowing pro-inflammatory microbial taxa to expand [115]. These studies highlight the role of bile acids in shaping the GI microbiota.

The microbiota can also be shaped by the host immune system. This effect is mostly limited to stratification and compartmentalisation of bacteria to avoid opportunistic invasion of host tissue, whilst species-specific effects are less probable due to the high amount of functional redundancy within the microbiota [52,116–119]. Both host-derived and administered antimicrobials play a key role in shaping the gut microbiota. In the GI tract, Paneth cells produce antimicrobials such as angiogenin 4, α-defensins, cathelicidins, collectins, histatins, lipopolysaccharide (LPS)-binding protein, lysozymes, secretory phospholipase A2 and lectins such as REGIIIα/γ [120]. These proteins are localised in the mucus layer and are virtually absent from the lumen, probably either due to poor diffusion through mucus or luminal degradation [51,121]. Many secreted antimicrobial proteins (AMPs) kill bacteria through direct interaction with, and disruption of the bacterial cell wall or inner membrane via enzymatic attack [51]. Reduced mucosal α-defensin expression has been demonstrated in patients with ileal Crohn's disease (CD), highlighting the importance of these proteins [122,123]. Secretory IgA (SIgA), another component of the immune system, co-localises with gut bacteria in the outer mucus layer and assists in limiting the exposure of the epithelial cell surface to bacteria [120,124]. SIgA is proposed to mediate bacterial biofilm formation via binding to SIgA receptors on bacteria [125]. The expression of SIgA receptors by bacteria is reduced in IgA-deficient individuals [126]. Dysbiosis of the microbiota, in particular an over-representation of segmented filamentous bacteria (SFB), occurs in mice deficient in IgA, an effect that may be particularly damaging to the host due to the ability of SFB to strongly adhere to the epithelium and activate the immune system [127].

Several environmental factors have been implicated in shaping the microbiota including geographical location, surgery, smoking, depression and living arrangements (urban or rural) [24,128–130]. Xenobiotics, such as antibiotics but not host-targeted drugs, shape the physiology and gene expression of the active human gut microbiome [131]. Antibiotic treatment dramatically disrupts both short- and long-term microbial balance, including decreases in the richness and diversity of the community. Clindamycin [132], clarithromycin and metronidazole [47], and ciproflaxin [33] have all been demonstrated to affect the microbiota structure for varying lengths of time. The exact effects and the time for recovery of the microbiota following antibiotic administration appear to be individual-dependent, a likely effect of the inter-individual variation in the microbiota prior to treatment [33,47,132]. An explorative study in humans showed that the administration of β-lactam intravenous therapy consisting of ampicillin, sulbactam and cefazolin affects both the microbial ecology and the production of key metabolites, such as acetyl phosphate and acetyl-CoA, that are involved in major cellular functions [133]. Recent investigations in mice demonstrated that microbiota depletion by antibiotics affected secondary bile acid and serotonin metabolism in the colon, resulting in delayed GI motility [134]. Antibiotic-treated mice are also more susceptible to pathogenic infection by antibiotic-associated pathogens, *S.* Typhimurium and *C. difficile*, due to an alteration in mucosal carbohydrate availability favouring their expansion into the gut [135]. A better understanding of the mechanisms leading to the antibiotic-induced blooms of bacteria and the biochemical activities and metabolites affected will help develop complementary and/or alternative strategies required for maintaining human health.

## Role of the GI microbiota in health

Owing to its large genomic content and metabolic complement, the gut microbiota provides a range of beneficial properties to the host. Some of the most important roles of these microbes are to help to maintain the integrity of the mucosal barrier, to provide nutrients such as vitamins or to protect against pathogens. In addition, the interaction between commensal microbiota and the mucosal immune system is crucial for proper immune function.

Colonic bacteria express carbohydrate-active enzymes, which endow them with the ability to ferment complex carbohydrates generating metabolites such as SCFAs [136]. Three predominant SCFAs, propionate, butyrate and acetate, are typically found in a proportion of 1:1:3 in the GI tract [137]. These SCFAs are rapidly absorbed by epithelial cells in the GI tract where they are involved in the regulation of cellular processes such as gene expression, chemotaxis, differentiation, proliferation and apoptosis [138]. Acetate is produced by most gut anaerobes, whereas propionate and butyrate are produced by different subsets of gut bacteria following distinct molecular pathways [102]. Butyrate is produced from carbohydrates via glycolysis and acetoacetyl-CoA, whereas two pathways, the succinate or propanediol pathway, are known for the formation of propionate, depending on the nature of the sugar [102]. In the human gut, propionate is mainly produced by Bacteroidetes, whereas the production of butyrate is dominated by Firmicutes [102,139,140]. For example, fermentation of starch by specialist Actinobacteria and Firmicutes, e.g. *Eubacterium rectale* or *E. hallii*, is thought to contribute significantly to butyrate production in the colon both directly and via metabolic cross-feeding [102]. *A. muciniphila* is a key propionate producer specialised in mucin degradation [141]. Propionate is primarily absorbed by the liver, whilst acetate is released into peripheral tissues [142]. The role of SCFAs on human metabolism has recently been reviewed [140,143]. Butyrate is known for its anti-inflammatory and anticancer activities [140,143]. Butyrate is a particularly important energy source for colonocytes [138]. A decreasing gradient of butyrate from lumen to crypt is suggested to control intestinal epithelial turnover and homeostasis by promoting colonocyte proliferation at the bottom of crypts, whilst increasing apoptosis and exfoliation of cells closer to the lumen [144]. Butyrate can attenuate bacterial translocation and enhance gut barrier function by affecting tight-junction assembly and mucin synthesis [140]. SCFAs also appear to regulate hepatic lipid and glucose homeostasis via complementary mechanisms. In the liver, propionate can activate gluconeogenesis, whilst acetate and butyrate are lipogenic [140]. SCFAs also play a role in regulating the immune system and inflammatory response [140]. They influence the production of cytokines, for example, stimulating the production of IL-18, an interleukin involved in maintaining and repairing epithelial integrity [138]. Butyrate and propionate are histone deacetylase inhibitors that epigenetically regulate gene expression [140,143]. SCFAs have also been shown to modulate appetite regulation and energy intake via receptor-mediated mechanisms [145]. Propionate has beneficial effects in humans acting on β-cell function [146] and attenuating reward-based eating behaviour via striatal pathways [147]. Microbial metabolites other than SCFAs have been reported to have an impact on intestinal barrier functions, epithelium proliferation and the immune system [148].

The GI microbiota is also crucial to the *de novo* synthesis of essential vitamins which the host is incapable of producing [149]. Lactic acid bacteria are key organisms in the production of vitamin B12, which cannot be synthesised by either animals, plants or fungi [149,150]. *Bifidobacteria* are main producers of folate, a vitamin involved in vital host metabolic processes including DNA synthesis and repair [151]. Further vitamins, which gut microbiota have been shown to synthesise in humans, include vitamin K, riboflavin, biotin, nicotinic acid, panthotenic acid, pyridoxine and thiamine [152]. Colonic bacteria can also metabolise bile acids that are not reabsorbed for biotransformation to secondary bile acids [113]. All of these factors will influence host health. For example, an alteration of the co-metabolism of bile acids, branched fatty acids, choline, vitamins (i.e. niacin), purines and phenolic compounds has been associated with the development of metabolic diseases such as obesity and type 2 diabetes [153].

There are many lines of evidence in support of a role for the gut microbiota in influencing epithelial homeostasis [7]. Germ-free mice exhibit impaired epithelial cell turnover which is reversible upon colonisation with microbiota [154]. A role has been demonstrated for bacteria in promoting cell renewal and wound healing, for example, in the case of *Lactobacilli rhamnosus* GG [155]. Furthermore, several species have been implicated in promoting epithelial integrity, such as *A. muciniphila* [156] and *Lactobacillus plantarum* [157]. In addition to modulating epithelial properties, bacteria are proposed to modulate mucus properties and turnover. Mice housed under germ-free conditions have an extremely thin adherent colonic mucus layer, but when exposed to bacterial products (peptidoglycan or LPS), the thickness of the adherent mucus layer can be restored to levels observed in conventionally reared mice [158]. *B. thetaiotaomicron* and *F. prausnitzii* have been implicated in the co-ordination of mucus production [159]. *R. gnavus* E1, *Lactobacillus casei* DN-114 001 and *B. thetaiotaomicron* are able to remodel mucin glycosylation, for example, by modulating glycosyltransferase expression [160–162]. It is proposed that these functions mediate the ability of other commensals or pathogens to colonise, potentially giving some commensal species a competitive advantage in the gut [162].

The GI microbiota is also important for the development of both the intestinal mucosal and systemic immune system as demonstrated by the deficiency in several immune cell types and lymphoid structures exhibited by germ-free animals. A major immune deficiency exhibited by germ-free animals is the lack of expansion of CD4+ T-cell populations. This deficiency can be completely reversed by the treatment of GF mice with polysaccharide A from the capsule of *B. fragilis* [163]. This process is mainly performed via the pattern recognition receptors (PRRs) of epithelial cells, such as Toll-like or Nod-like receptors, which are able to recognise the molecular effectors that are produced by intestinal microbes. These effectors mediate processes that can ameliorate certain inflammatory gut disorders, discriminate between beneficial and pathogenic bacteria or increase the number of immune cells or PRRs [164]. SFB, a class of anaerobic and clostridia-related spore-forming commensals present in the mammalian GI tract, actively interact with the immune system [165]. Unlike other commensal bacteria, SFB are closely associated with the epithelial lining of the mammalian GI tract membrane, which stimulates epithelial cells to release serum amyloid A1 [148]. Colonisation with SFB may also direct post-natal maturation of the gut mucosal lymphoid tissue, trigger a potent and broad IgA response, stimulate the T-cell compartment and up-regulate intestinal innate defence mediators, suggesting immune-stimulatory capacities of SFB (as reviewed in [143]). *A. muciniphila* has been correlated with protection against several inflammatory diseases [84,87,166–170], suggesting that this strain possesses anti-inflammatory properties although the underlying mechanisms have not been completely elucidated [171]. Individuals with CD display mucosal dysbiosis characterised by reduced diversity of core microbiota and lower abundance of *F. prausnitzii* [172]. *F. prausnitzii* monitoring may therefore serve as a biomarker to assist in gut disease diagnostics [173]. Recently, an anti-inflammatory protein from *F. prausnitzii* was shown to inhibit the NF-κB pathway in intestinal epithelial cells and prevent colitis in an animal model [174].

The physical presence of the microbiota in the GI tract also influences pathogen colonisation by, for example, competing for attachment sites or nutrient sources, and by producing antimicrobial substances [9]. Antibiotics have a profound impact on the microbiota that alter the nutritional landscape of the gut and lead to the expansion of pathogenic populations [175]. For example, *S.* Typhimurium and *C. difficile* utilise fucose and sialic acid liberated by the gut microbiota, and increasing sialic acid levels post-antibiotic treatment favour their expansion within the gut [135]. Enterohaemorrhagic *E. coli* has also been shown to access fucose or sialic acid liberated by the gut microbiota from mucins [176]. Dietary fibre deficiency, together with a fibre-deprived, mucus-eroding microbiota, promotes greater epithelial access and lethal colitis by the mucosal pathogen *Citrobacter rodentium* in mice [83]. The GI microbiota, via its structural components and metabolites, also stimulates the host to produce various antimicrobial compounds. These include AMPs such as cathelicidins, C-type lectins and (pro)defensins by the host Paneth cells via a PRR-mediated mechanism [51]. The other mechanism by which the gut microbiota can limit pathogen overgrowth is by inducing mucosal SIgA [177]. Induction of SIgAs directed against gut commensal bacteria occurs via an M-cell-mediated sampling mechanism [178]. SIgAs are then anchored in the outer layer of colonic mucus through combined interactions with mucins and gut bacteria, thus providing immune protection against pathogens whilst maintaining a mutually beneficial relationship with commensals [124]. PRR–MAMP (pattern recognition receptor–microbe-associated molecular patterns) cross-talk results in activation of several signalling pathways that are essential for promoting mucosal barrier function and production of AMPs, mucins and IgA, contributing to host protection against invading pathogens and preventing the overgrowth of the commensals themselves [179].

## Conclusion

Given the close symbiotic relationship existing between the gut microbiota and the host, it is not surprising to observe a divergence from the normal microbiota composition (generally referred to as dysbiosis) in a plethora of disease states ranging from chronic GI diseases to neurodevelopmental disorders [12, 180]. The application of metabolomics approaches has greatly advanced our understanding of the mechanisms linking the gut microbiota composition and its activity to health and disease phenotypes. At a functional level, a potential way to describe a ‘dysbiotic microbiota’ might be one which fails to provide the host with the full complement of beneficial properties. Whether dysbiosis of the microbiota is a cause or a consequence of the disease is therefore likely to exacerbate the progression of the disease and affect the type of strategies needed to restore symbiosis. Depending on the type and stage of disease, these include the development of microbiome modulators (e.g. antimicrobials, diet, prebiotics or probiotics) mostly aimed at changing the composition of the host microbiota, or of microbial-based solutions to replace some of the defective microbes and their associated benefits (e.g. specific commensal strains, probiotics, defined microbial communities, microbial-derived signalling molecules or metabolites). Given the contribution of host genetics in many diseases associated with a dysbiotic microbiota, dual therapeutic strategies (e.g. combining immunotherapy and microbiota-targeted approaches) may also be required to restore the environment required to re-establish an effective communication between the host and the targeted microbiota. Success in these endeavours is dependent on our mechanistic understanding of how the microbiota affects and is affected by the host at a molecular and biochemical level.

## Abbreviations

AMPs: antimicrobial proteins
CD: Crohn's disease
FUT2: α1,2-fucosyltransferase
GHs: glycoside hydrolases
GI: gastrointestinal
IBD: inflammatory bowel disease
IBS: inflammatory bowel syndrome
IL-18: interleukin
LPS: lipopolysaccharide
MACs: microbiota-accessible carbohydrates
PLs: polysaccharide lyases
PRRs: pattern recognition receptors
rRNA: ribosomal RNA
SCFA: short-chain fatty acid
SFB: segmented filamentous bacteria
SIgA: secretory IgA
